# Non-esterified Fatty Acid Induce Dairy Cow Hepatocytes Apoptosis via the Mitochondria-Mediated ROS-JNK/ERK Signaling Pathway

**DOI:** 10.3389/fcell.2020.00245

**Published:** 2020-04-28

**Authors:** Yu Li, Hongyan Ding, Leihong Liu, Yuxiang Song, Xiliang Du, Shibin Feng, Xichun Wang, Xiaobing Li, Zhe Wang, Xinwei Li, Jinchun Li, Jinjie Wu, Guowen Liu

**Affiliations:** ^1^College of Animal Science and Technology, Anhui Agricultural University, Hefei, China; ^2^Key Laboratory of Zoonosis, Ministry of Education, College of Veterinary Medicine, Jilin University, Changchun, China

**Keywords:** NEFA, apoptosis, mitochondria-mediated ROS-JNK/ERK signaling pathway, dairy cow, hepatocyte

## Abstract

Elevated plasma non-esterified fatty acid (NEFA) levels and hepatocytes damage are characteristics of ketosis in dairy cows. Oxidative stress is associated with the pathogenesis of NEFA-induced liver damage. However, the exact mechanism by which oxidative stress mediates NEFA-induced hepatocytes apoptosis and liver injury remains poorly understood. The results of the present study demonstrated that NEFA contribute to reactive oxygen species (ROS) generation, resulting in an imbalance between oxidative and antioxidant species, transcriptional activation of p53, transcriptional inhibition of nuclear factor E2-related factor 2 (Nrf2), loss of mitochondria membrane potential (MMP) and release of apoptosis-inducing factor (AIF) and cytochrome *c* (cyt *c*) into the cytosol, leading to hepatocytes apoptosis. Besides, NEFA triggered apoptosis in dairy cow hepatocytes via the regulation of c-Jun N-terminal kinase (JNK), extracellular signal-regulated protein kinases 1 and 2 (ERK1/2), Bcl-2-associated X protein (Bax), B-cell lymphoma gene 2 (Bcl-2), caspase 9 and poly (ADP-ribose) polymerase (PARP). Pretreatment with the inhibitor SP600125 or PD98059 or the antioxidant *N*-acetylcysteine (NAC) revealed that NEFA-ROS-JNK/ERK-mediated mitochondrial signaling pathway plays a crucial role in NEFA-induced hepatocytes apoptosis. Moreover, the results suggested that the transcription factors p53 and Nrf2 function downstream of this NEFA-ROS-JNK/ERK pathway and are involved in NEFA-induced hepatocytes apoptosis. In conclusion, these findings indicate that the NEFA-ROS-JNK/ERK-mediated mitochondrial pathway plays an important role in NEFA-induced dairy cow hepatocytes apoptosis and strongly suggests that the inhibitors SP600125 and PD98059 and the antioxidant NAC may be developed as therapeutics to prevent hyperlipidemia-induced apoptotic damage in ketotic dairy cows.

## Highlights

- NEFA could induce oxidative stress and apoptosis in hepatocytes of cows.
- NEFA activated mitochondria-mediated ROS-JNK/ERK pathway to induce cell apoptosis.
- NEFA increased the transcriptional activation of p53 and inhibited Nrf2.

## Introduction

In dairy cows, ketosis is a metabolic disorder that occurs primarily during the transition period (21 days before and after parturition) (Melendez et al., 2006; Li et al., 2012). During the transition period, dairy cows experience a state of negative energy balance (NEB) induced by a low intake of dry matter and an increased demand for energy to support milk production (Li et al., 2015; Zhu et al., 2019). The NEB initiates fat mobilization and a subsequent increase in blood non-esterified fatty acid (NEFA) concentration. Large quantities of NEFA are metabolized into ketones in hepatocytes, thereby inducing ketosis (Li et al., 2016a). Therefore, a high concentration of NEFA is an important pathogenic factor that may be associated with the pathogenicity of some NEB-related metabolic disorders, such as ketosis and fatty liver. Clinical investigations have demonstrated that dairy cows with ketosis or with fatty liver display oxidative stress. Several lines of evidence *in vivo* and *in vitro* have demonstrated that a high-fat diet or high levels of NEFA induce oxidative stress, resulting in hepatocytes injury. Because of the NEB, the lipotoxicity of NEFA is due primarily to reactive oxygen species (ROS) production during mitochondrial oxidation (Schönfeld and Wojtczak, 2008). Continuous, incomplete NEFA oxidation results in an abundance of oxygen radicals in the liver that induce hepatocytes damage. Besides, *in vivo* study indicated that ketotic cows displayed hepatic oxidative stress and apoptotic damage (Du et al., 2017). However, the underlying mechanism by which elevated NEFA levels induce hepatocytes injury remains unknown.

High levels of NEFA oxidation increase mitochondrial ROS generation, which has been observed in a variety of cells, including rat hepatoma cells, skeletal muscle cells, cardiomyocytes, vascular smooth muscle cells, and adipocytes (Lu et al., 1998; Guo et al., 2006; Fauconnier et al., 2007; Rachek et al., 2007; Srivastava et al., 2007). ROS induce secondary “hits” to the hepatocytes, such as lipid peroxidation and DNA damage, ultimately causing hepatocytes apoptosis (Cui et al., 2019).

Previous studies have demonstrated that members of the mitogen-activated protein kinase (MAPK) family of Ser/Thr-type kinases play an essential role in apoptosis (Chang and Karin, 2001). The three major MAPKs are c-Jun N-terminal kinase (JNK), extracellular signal-regulated kinase (ERK1/2) and p38 mitogen-activated protein kinase (p38 MAPK) (Shen et al., 2013). JNK is thought to be involved in the induction of apoptosis by activating cellular damage signaling (Chen et al., 2014). ERK may participate in cellular differentiation, survival and death (Chang and Karin, 2001). Moreover, ROS may participate in the alteration of MAPK expression (Thannickal and Fanburg, 2000). ROS activated JNK and p38 MAPK phosphorylation in berberine- and free fatty acid (FFA)-induced SW620 cells, triggered bovine hepatocytes apoptosis and downregulated ERK phosphorylation in response to 4-OHE_2_-induced cell death (Chen et al., 2005; Hsu et al., 2007; Song et al., 2014). Additionally, the changes in p38 MAPK, JNK, and ERK that are induced by many stimulus are involved in the transcription or phosphorylation of p53 and nuclear factor E2-related factor 2 (Nrf2) (Cheng et al., 2008; Sun et al., 2009).

Moreover, the phosphorylation of p53, which is mediated by JNK, plays an important role in pro-apoptotic activity (Sanchez-Prieto et al., 2000). It has also been reported that oxidative stress–associated JNK/ERK-mediated apoptosis requires inhibition of the transcription factor Nrf2 in various cell types (Cardaci et al., 2010). P53 activation and Nrf2 inhibition trigger several signaling pathways that may lead to cell cycle arrest, apoptosis, DNA repair and mitochondrial dysfunction (Marchenko et al., 2000; Bonini et al., 2004; Bae et al., 2005; Endo et al., 2006). As a transcription factor, the tumor suppressor p53 downregulates anti-apoptotic Bcl-2 proteins and trans-activates the pro-apoptotic protein Bax to activate the mitochondrial apoptosis pathway, which is characterized by the permeability of the inner mitochondrial membrane and by the loss of the mitochondrial membrane potential (MMP) (Sharma et al., 2012). Previous reports have shown that p53 inhibits the transcriptional activation of Nrf2 target genes in mouse Hepal-6 cells (Faraonio et al., 2006). Inhibiting Nrf2 is critical to p53-mediated apoptosis pathway activation (Iida et al., 2007). Moreover, the inhibition of Nrf2, which is involved in regulating numerous antioxidant genes related to oxidative stress and apoptosis, is mediated by Bcl-2 family proteins (Niture and Jaiswal, 2011, 2012).

The mitochondrial, or intrinsic, apoptosis pathway is induced via the deprivation of growth and differentiation factors as environmental stimuli. These stimulus promote the translocation of pro-apoptotic proteins, such as Bax, to mitochondria, altering the integrity of these organelles. Subsequently, various apoptotic effectors that can activate the cell death machinery, such as cytochrome c (cyt *c*) and apoptosis-inducing factor (AIF), are released from mitochondria (Salvesen and Duckett, 2002).

Dairy cow with ketosis or fatty liver displays a high blood concentration of NEFA, oxidative stress and liver injury. Liver injury in dairy cows with ketosis or fatty liver may be associated with NEFA-mediated oxidative stress. Thus, elucidating the mechanisms by which NEFA induce oxidative stress in dairy cows with ketosis is of great interest. Therefore, this study aimed to investigate the molecular mechanism underlying NEFA-induced liver oxidative damage *in vitro*. Our studies demonstrated that NEFA induce dairy cow hepatocytes apoptosis by increasing intracellular ROS generation, JNK/ERK activation, p53/Nrf2-mediated transcription and mitochondrial dysfunction.

## Materials and Methods

### Hepatocytes Culture

A female neonatal calf was anesthetized using thiamylal sodium and the caudate lobe was removed. As described in previous studies, hepatocytes were harvested using a two-step collagenase perfusion method (Li et al., 2016b, 2019). The cell density was adjusted to 2 × 10^6^ cells/ml in adherent culture medium containing 10% calf serum, 10^–6^ mol/L insulin, 10^–6^ mol/L DEX, 10 μg/ml V_c_, 100 U/ml benzylpenicillin, and 100 μg/ml streptomycin and the hepatocytes suspensions were seeded on 6-well tissue culture plates (2 ml/well). The hepatocytes were incubated at 37°C in 5% CO_2_. After a 4-h attachment period, the medium and unattached cells were removed and replaced with growth medium containing 5% fetal bovine serum (FBS). Every 24 h, the medium was changed to fresh medium. The cultured hepatocytes were observed every day under a phase-contrast microscope and the adherence status and morphological changes were evaluated.

### Cell Viability Assay

Cell viability was assessed using the cholecystokinin octapeptide (CCK-8) assay according to the manufacturer’s instructions (Beyotime Institute of Biotechnology, China). Primary cultured dairy cow hepatocytes were seeded on 96-well plates at a density of 2 × 10^4^ cells/well (100 μL of medium per well; 5 wells per treatment condition). Next, the hepatocytes were cultured for 72 h at 37°C in 5% CO_2_. The hepatocytes were treated with various NEFA concentrations for 4 h (Shi et al., 2015). Then, 10 μL of CCK-8 solution was added to each well, followed by incubation for 3.5 h. The absorbance at 570 nm was measured using a microplate reader (Bio-Tec, CA, United States).

### Measurement of the LDH, Glutathione (GSH), and MDA Levels

To determine cell death and intracellular redox status, the GSH and MDA contents and LDH release from hepatocytes were measured using a cytotoxicity detection kit (Roche Applied Science, Shanghai, China) according to the manufacturer’s protocol. The hepatocytes were grown in a six-well plate and treated with various NEFA concentrations for 4 h. The cultured supernatants were collected via centrifugation at 4,000 rpm for 10 min. LDH activity and the GSH and MDA contents in the supernatants were determined using an automated biochemical analyzer (Hitachi, Japan).

### Hoechst 33258 Staining Assay

The characteristics of apoptosis, including DNA condensation and nuclear fragmentation, were observed via Hoechst 33258 staining according to the manufacturer’s instructions (Beyotime Institute of Biotechnology, China). The hepatocytes were cultured on coverslips at a density of 1 × 10^5^ cells/well in 24-well plates in the presence of various NEFA concentrations or 4 h. The cells were stained with 0.5 ml Hoechst 33258 for 5 min and then observed under a fluorescence microscope (Olympus Optical Co., Ltd., Tokyo, Japan).

### Determination of Apoptosis

Hepatocytes apoptosis was determined via Annexin V-FITC and propidium iodide (PI) double staining using a FITC Annexin V Apoptosis Detection Kit I (Becton-Dickinson, CA, United States) according to the manufacturer’s instructions. The hepatocytes (2 × 10^6^ cells/well in a 6-well plate) were treated with various NEFA concentrations at 37°C in the presence or absence of SP600125, PD98059, and N-acetylcysteine (NAC). Nuclear staining was immediately observed using a confocal laser scanning microscope (CLSM).

### Measurement of ROS

As the previous study described, the levels of intracellular ROS were determined using the peroxide-sensitive fluorescent probe 2’7’-dichlorofluorescein diacetate (DCFH-DA) (Zhang et al., 2019). After treatment in NEFA for 4 h, the cells were collected and washed twice with PBS. The cells were exposed to serum-free medium containing 10 mM DCFH-DA in the dark for 30 min and then washed three times with cold PBS. The fluorescence was measured via flow cytometry (FACSCalibur, Becton-Dickinson, Sunnyvale, CA, United States).

### MMP Assay

The MMP was measured by staining with JC-1 (Beyotime Institute of Biotechnology, Nanjing, China) according to the manufacturer’s instructions. The hepatocytes were cultured on coverslips. After treatment with various NEFA concentrations for 4 h, 1 ml JC-1 dye was added to the 6-well plate, and the cells were incubated for 20 min at 37°C. Then, the hepatocytes were washed with JC-1 dye buffer solution (1×) twice and immediately observed using a CLSM. Red fluorescence indicates the aggregation of JC-1 in mitochondria, and green fluorescence indicates the appearance of JC-1 monomers in the cytoplasm because of mitochondrial depolarization.

### Western Blot Analysis

The cells were washed with ice-cold PBS, including centrifugation at 800 *g* for 5 min, and were resuspended in 200 μL of RIPA cell lysis buffer (1% Triton X-100, 1% sodium deoxycholate, 0.1% SDS, 1 mM protease inhibitor and 10 mM phosphatase inhibitor), completely vortexed and incubated on ice for 20 min. The protein extracts were centrifuged at 14,000 *g* for 10 min at 4°C, and the supernatants were collected for analysis. The protein concentrations were determined using a bicinchoninic acid (BCA) protein assay kit (Applygen Technologies, Inc., Beijing, China). Then, 50 μg of protein was separated via 10% SDS-polyacrylamide gel electrophoresis and transferred to a polyvinylidene difluoride (PVDF) membrane. After being blocked with 3% bovine serum albumin (BSA) for 4 h at room temperature, the membranes were incubated with primary antibodies at 4°C overnight. The membranes were washed four times and then incubated with horseradish peroxidase (HRP)-conjugated secondary antibody at room temperature for 45 min. The protein bands were visualized using enhanced chemiluminescence (ECL) and imaged using the alpha ultrasensitive FluorChem E chemiluminescence imaging system (ProteinSimple, Santa Clara, CA, United States).

### Preparation of Subcellular Protein Fractions

The hepatocytes were treated with various NEFA concentrations for 4 h following pretreatment with the antioxidant NAC or with an inhibitor of JNK (SP600125) or ERK (PD98059). To detect the redistribution of cyt *c* and AIF, the proteins in the cytosolic fraction were isolated using a cytosol protein extraction kit (Beyotime Institute of Biotechnology, Nanjing, China) according to the multiple centrifugation method. The protein concentrations were measured using the BCA method (Applygen, Beijing, China).

### Immunofluorescence

The hepatocytes were cultured on glass coverslips and treated in various NEFA concentrations for 4 h. Cells were washed three times with serum-free medium and fixed using 4% paraformaldehyde at room temperature for 30 min. After washing three times with PBS, cells were permeabilized using 0.1% Triton X-100 for 10 min. Then, the hepatocytes were incubated in a mouse anti-p53 antibody (1:500) or a rabbit anti-Nrf2 antibody (1:200) at 4°C overnight, followed by incubation in the appropriate secondary antibody (1:200) for 45 min and counterstained with Hoechst 33258 (Beyotime Institute of Biotechnology, Nanjing, China). Finally, the coverslips were sealed using glycerol, and the samples were observed via laser confocal microscopy (FV500, Olympus, China).

### Electrophoretic Mobility Shift Assay (EMSA)

Briefly, the nuclear proteins were extracted, and the protein concentrations were measured using Bio-Rad protein assay reagent (Bio-Rad, Munich, Germany). The p53 and Nrf2 probes were labeled with biotin for 30 min at 37°C. Equal amounts of 4 μg of nuclear protein from each sample were included in the binding reaction for 20 min at room temperature using a Lightshift EMSA Optimization and Control Kit (Pierce Biotechnology, Inc., Rockford, IL, United States) according to the manufacturer’s instructions. The protein-DNA complexes were separated using non-denaturing 6.5% polyacrylamide TBE electrophoresis gels and were transferred to nylon membranes. The biotin-labeled probes were detected using chemiluminescence solutions (Pierce Biotechnology, Inc., Rockford, IL, United States). The labeled bands were measured using a Syngene G:BOX Chemi XR5 gel imager (Syngene, Cambridge, United Kingdom).

### Statistical Analysis

The experiments were performed at least five times, and statistical analysis was performed using Statistical Package for the Social Sciences (SPSS) 16.0 software (SPSS, Inc., Chicago, IL, United States). The data, which are expressed as means ± standard deviations (SD) and multiple comparison was carried out via a one-way analysis of variance or Student’s *t*-test. A *P*-value < 0.05 was considered statistically significant.

## Results

### NEFA Induced Hepatocytes Oxidative Stress and Apoptosis

**FIGURE 1:**
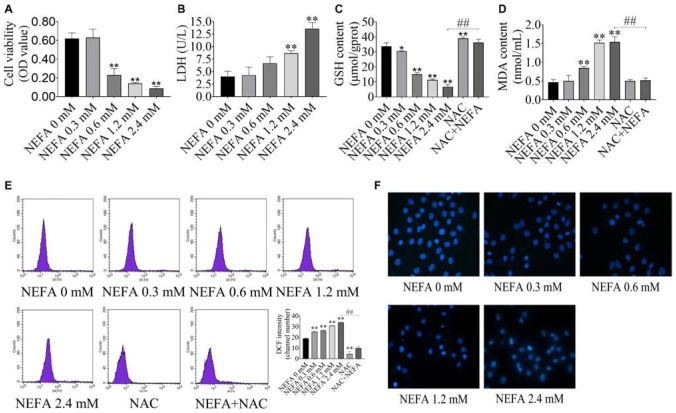
NEFA induces oxidative stress and apoptosis in dairy cow hepatocytes. **(A)** cell survival of hepatocytes treated with various NEFA concentrations (0, 0.3, 0.6, 1.2, or 2.4 mM) for 4 h was determined via the CCK-8 assay. **(B)** Cytotoxicity of hepatocytes treated with various NEFA concentrations (0, 0.3, 0.6, 1.2, or 2.4 mM) for 4 h was measured using an LDH assay kit. **(C)** The GSH content, **(D)** the MDA content, and **(E)** intracellular ROS production in dairy hepatocyes incubated with different concentrations of NEFA (0, 0.3, 0.6, 1.2, or 2.4 mM) without or with NAC (10 mM). **(F)** Cellular apoptosis in dairy hepatocyes incubated with different concentrations of NEFA (0, 0.3, 0.6, 1.2, or 2.4 mM) were stained with Hoechst 33258 and were observed under a fluorescence microscope. The data are presented as the mean ± SD of three independent experiments. *p < 0.05 and **p < 0.01 vs. the control group. ^#^*p* < 0.05 and ^##^*p* < 0.01 vs. 2.4 mM NEFA-treated group.

To determine whether NEFA induce hepatocytes cytotoxicity, the effect of NEFA on cell viability was evaluated. Hepatocytes were treated with various concentrations of NEFA (0–2.4 mM) for 4 h. The results indicated that NEFA inhibited hepatocytes growth and increased LDH release from hepatocytes in a dose-dependent manner (Figures 1A,B). Cell viability dramatically decreased in the 0.6, 1.2, and 2.4 mM NEFA treatment groups. LDH release significantly increased in the 1.2 and 2.4 mM NEFA treatment groups (Figure 1B). GSH is an intracellular antioxidant that protects against oxidative stress by scavenging ROS. In the present study, we determined the levels of intracellular GSH in NEFA-treated cells in the presence or absence of NAC. As shown in Figure 1C, the GSH content decreased gradually and was significantly reduced in the 0.6, 1.2, and 2.4 mM NEFA treatment groups. MDA is an indicator of lipid peroxidation caused by oxidative stress. We found that NEFA enhanced MDA production, which markedly increased in the 0.6, 1.2, and 2.4 mM NEFA treatment groups (Figure 1D). The results also revealed that NAC markedly promoted GSH production and significantly inhibited the NEFA-induced MDA production (Figures 1C,D). The intracellular ROS levels in hepatocytes were analyzed via DCFH-DA staining, revealing a NEFA dose-dependent increase in intracellular ROS levels, which were significantly higher in the 0.3, 0.6, 1.2, and 2.4 mM NEFA treatment groups compared with the control group (Figure 1E). However, the antioxidant NAC inhibited this NEFA-mediated increase in ROS. These results further confirmed that NEFA induces redox imbalance in dairy cow hepatocytes. Hoechst 33258-stained cells were observed via fluorescence microscopy and displayed chromatin condensation and fragmentation in the 1.2 and 2.4 mM NEFA-treated groups, which was distinct from the properties of chromatin in the control group (Figure 1F). These results indicated that NEFA induces apoptosis in hepatocytes via a ROS-dependent pathway.

### The ERK/JNK Pathway Is Involved in NEFA-Induced Dairy Cow Hepatocytes Apoptosis

**FIGURE 2:**
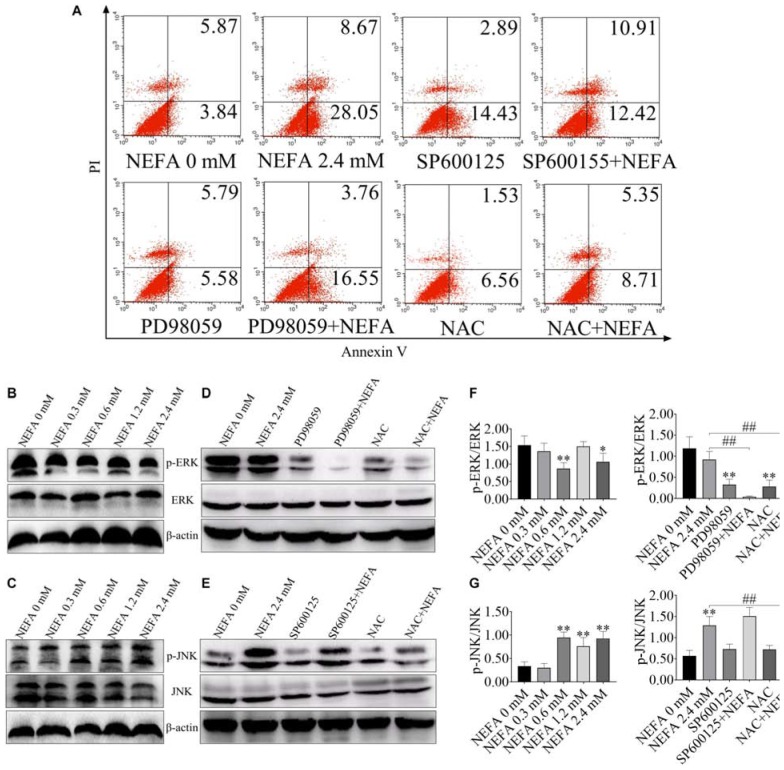
Effect of NEFA on the apoptosis signaling pathway in hepatocytes. **(A)** Apoptotic cells analysis in dairy cows hepatocytes incubated without or with NEFA (2.4 mM) in the absence or presence of SP600125 (10 μM), PD98059 (10 μM), and NAC (10 mM). The protein expression levels of **(B)** ERK and **(C)** JNK in dairy cows hepatocytes treated with various concentrations of NEFA (0, 0.3, 0.6, 1.2, or 2.4 mM). The protein expression levels of **(D)** ERK and **(E)** JNK in dairy cows hepatocytes incubated without or with NEFA (2.4 mM) in the absence or presence of SP600125 (10 μM), PD98059 (10 μM), and NAC (10 mM). Quantification of **(F)** p-ERK/ERK and **(G)** p-JNK/JNK (*n* = 5). *p < 0.05 and **p < 0.01 vs. the control group. ^#^*p* < 0.05 and ^##^*p* < 0.01 vs. 2.4 mM NEFA-treated group.

FITC-Annexin V/PI doubled staining was used to reveal the cell apoptosis. The percentages of apoptotic cells in the control, 2.4 mM NEFA-treated, SP600125-treated, SP600125- and 2.4 mM NEFA-treated, PD98059-treated, PD98059- and 2.4 mM NEFA-treated, NAC-treated and NAC- and 2.4 mM NEFA-treated groups were 9.71, 36.72, 17.32, 23.33, 11.37, 20.31, 8.09, and 14.06%, respectively (Figure 2A).

ERK and JNK are the main two MAPK family members that participate in cell proliferation, cell differentiation and apoptosis. In the present study, to consider whether ERK and JNK are involved in NEFA-induced hepatocytes apoptosis, the phosphorylation levels of ERK and JNK were measured in NEFA-treatment hepatocytes via western blot. As shown in Figures 2B–D,F, NEFA significantly inhibited the activation of ERK1/2 and increased the activation of JNK. PD98059 (an inhibitor of ERK) reduced the phosphorylation of ERK, whereas NAC (a ROS scavenger) increased ERK phosphorylation (Figures 2D,F). The JNK inhibitor SP600125 and NAC inhibited the NEFA-induced upregulation of the phosphorylation of JNK (Figures 2E,G). These results indicated that activation of JNK and inhibition of ERK were involved in NEFA-induced hepatocytes apoptosis.

**FIGURE 3:**
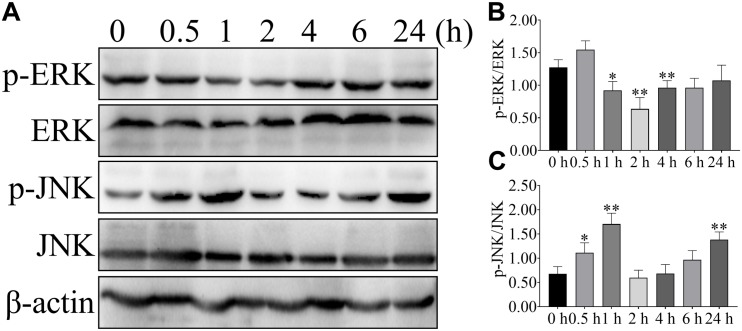
Effect of NEFA on the phosphorylation of ERK and JNK in hepatocyte at different time points. Hepatocytes were treated with 2.4 mM NEFA at 0, 0.5, 1, 2, 4, 6, and 24 h. Expression of **(A)** phosphorylation-ERK (p-ERK), ERK, phosphorylation-JNK (p-JNK) and JNK were evaluated by western blot. Quantification of **(B)** p-ERK/ERK and **(C)** p-JNK/JNK (*n* = 5). *p < 0.05 and **p < 0.01 vs. the control group.

To further confirm the effect of NEFA on the ERK and JNK, phosphorylation of ERK and JNK were detected after 2.4 mM NEFA treatment at different time points (Figure 3). Phosphorylation of ERK was greater reduced relative to control levels within 1 or 2 h after stimulation and return to control level by 4 h (Figures 3A,B). Stimulation with 2.4 mM NEFA induced elevated phosphorylated of JNK at 1 h relative to the control group. Signaling declined from 2 to 6 h post-stimulation, with an increase to high phosphorylation by 24 h (Figures 3A,C). These results indicated that fast and more extensive ERK and JNK alteration.

### Apoptosis-Related Genes Are Responsible for the NEFA-Induced Activation of the ROS-ERK/JNK Apoptosis Signaling Pathway

**FIGURE 4:**
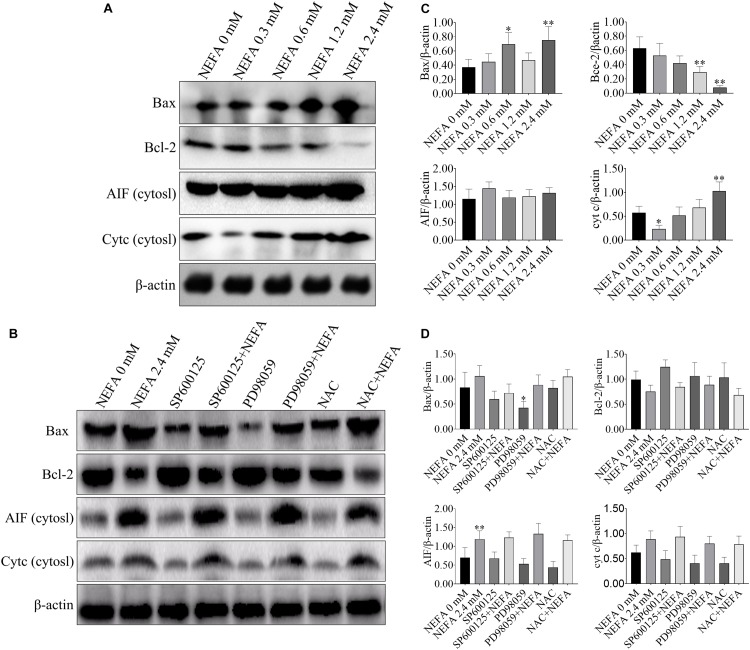
Effect of NEFA on the expression levels of Bax, Bcl-2, cyt *c*, and AIF in hepatocytes. **(A)** The protein expression of Bax, Bcl-2, cyt *c*, and AIF in cytosol in dairy cows hepatocytes treated with various concentrations of NEFA (0, 0.3, 0.6, 1.2, or 2.4 mM). **(B)** The protein expression of Bax, Bcl-2, cyt *c*, and AIF in cytosol in dairy cows hepatocytes incubated without or with NEFA (2.4 mM) in the absence or presence of SP600125 (10 μM), PD98059 (10 μM), and NAC (10 mM). Quantification of **(C)** Bax, Bcl-2, AIF, and cyt *c* in the absence of SP600125 (10 μM), PD98059 (10 μM), and NAC (10 mM). Quantification of **(D)** Bax, Bcl-2, AIF, and cyt *c* in the presence of SP600125 (10 μM), PD98059 (10 μM), and NAC (10 mM). All results were obtained from three independent experiments and are expressed as the mean ± SD. *p < 0.05 and **p < 0.01 vs. the control group. ^#^*p* < 0.05 and ^##^*p* < 0.01 vs. 2.4 mM NEFA-treated group.

Bcl-2 family proteins, which include both anti- and pro-apoptosis proteins, are crucial regulators of apoptosis, and a slight imbalance in their expression can result in the release of cyt *c* and other apoptotic factors from mitochondria into the cytosol and the downstream activation of the caspase cascade, leading to activation cell death (García-Sáez, 2012). To further confirm that whether NEFA can induce apoptosis, western blot was performed to determine the protein expression levels of Bax, Bcl-2, AIF and cyt *c*, caspase 3, caspase 9, PARP, apaf-1, and ASK1. As shown in Figures 4A,C, NEFA markedly increased the protein expression levels of Bax and cyt *c* (in the cytosol) but significantly decreased the protein expression of Bcl-2. However, the JNK inhibitor SP600125 and NAC attenuated the effects of NEFA on Bcl-2 family proteins and cyt *c*, whereas the ERK inhibitor PD98059 further enhanced these effects (Figures 4B,D).

**FIGURE 5:**
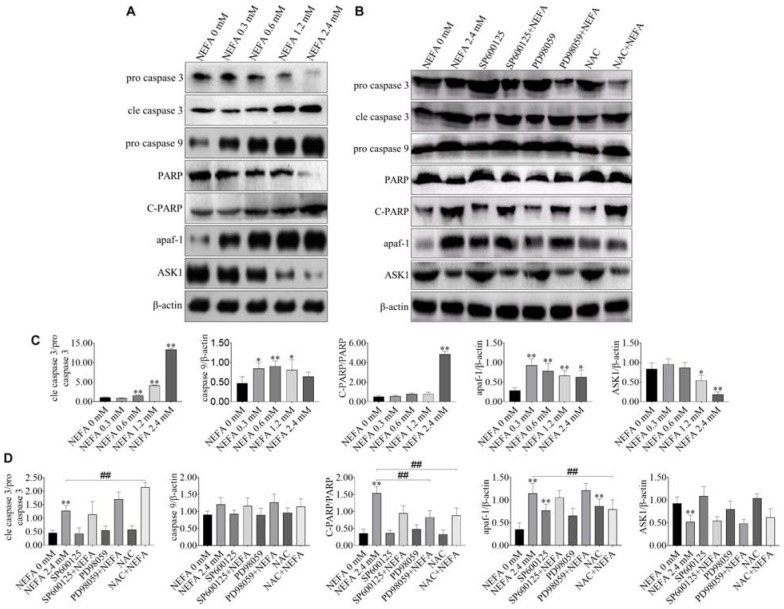
Effect of NEFA on apoptosis-related genes. **(A)** The protein expression levels of caspase 3, cleaved caspase 3, caspase 9, PARP, C-PARP, apaf-1, and ASK1 in dairy cows hepatocytes treated with various concentrations of NEFA (0, 0.3, 0.6, 1.2, or 2.4 mM). **(B)** The protein expression levels of caspase 3, cleaved-caspase 3, caspase 9, PARP, C-PARP, apaf-1, and ASK1 in dairy cows hepatocytes incubated without or with NEFA (2.4 mM) in presence of SP600125 (10 μM), PD98059 (10 μM), and NAC (10 mM). Quantification of **(C)** cleaved-caspase 3/caspase 3, caspase 9, C-PARP/PARP, apaf-1, and ASK1 in the absence of SP600125 (10 μM), PD98059 (10 μM), and NAC (10 mM). Quantification of **(D)** cleaved-caspase 3/caspase 3, caspase 9, C-PARP/PARP, apaf-1, and ASK1 in the presence of SP600125 (10 μM), PD98059 (10 μM), and NAC (10 mM). All results were obtained from three independent experiments and are expressed as the mean ± SD. *p < 0.05 and **p < 0.01 vs. the control group.

NEFA markedly upregulated the protein expression levels of cleaved caspase 3, cleaved PARP, pro-caspase 9, and apaf-1 but downregulated the protein expression levels of pro-caspase 3 and ASK1 (Figures 5A,C). The JNK inhibitor SP600125 and NAC inhibited the effects of NEFA on the caspase cascade, whereas the ERK inhibitor PD98059 enhanced these effects (Figures 5B,D). These results indicated that NEFA-induced dairy cow hepatocytes apoptosis occurs via the mitochondria-dependent activation of the ROS-ERK/JNK apoptosis signaling pathway.

### NEFA Cause a Loss of the MMP in Dairy Cow Hepatocytes

**FIGURE 6:**
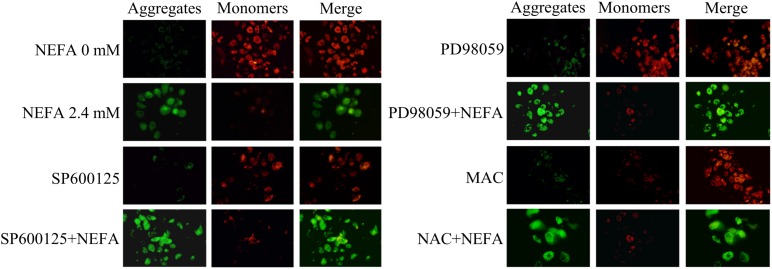
Effect of NEFA on the MMP in dairy cow hepatocytes. The MMP was assessed using the lipophilic cationic probe JC-1. The red signal indicates JC-1 aggregation in mitochondria. The green signal indicates cytosolic JC-1 monomers, reflecting a loss of the MMP. The groups are as follows: NEFA 0 mM; NEFA 2.4 mM; SP600125 group; SP600125 + NEFA group; PD98059 group; PD98059 + NEFA group; NAC group; NAC + NEFA group.

To further determine the mechanism underlying NEFA-induced apoptosis, the effect of NEFA on the MMP was evaluated via JC-1 dye staining and observation using a CLSM. As shown in Figure 6, NEFA significantly decreased the MMP in dairy cow hepatocytes. Pretreatment of hepatocytes with the JNK inhibitor SP600125 or NAC blocked the NEFA-induced loss of the MMP. However, the ERK inhibitor PD98059 enhanced this loss of the MMP. These results further indicate that NEFA induces mitochondrial damage, thereby activating the ROS-ERK/JNK apoptosis signaling pathway.

### NEFA Promote the Transcriptional Activity, Translocation, and Expression of p53 in Dairy Cow Hepatocytes

**FIGURE 7:**
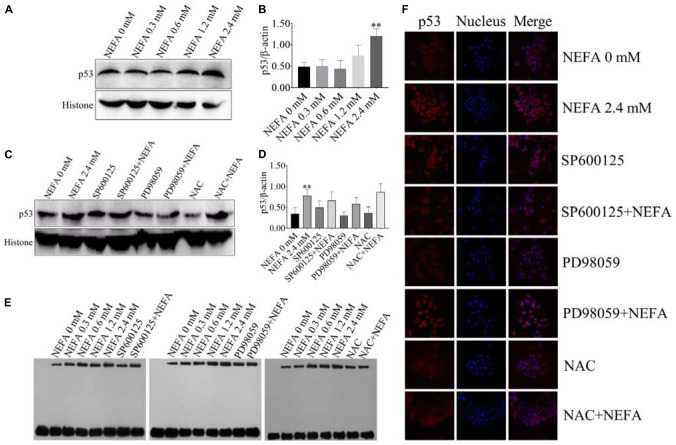
Effect of NEFA on the activation of p53. **(A)** The protein expression levels and **(B)** quantification of p53 in dairy cows heaptocytes incubated with different concentrations of NEFA (0, 0.3, 0.6, 1.2, or 2.4 mM). **(C)** The protein expression levels and **(D)** quantification of p53 in dairy cows heaptocytes incubated with different concentrations of NEFA (0, 0.3, 0.6, 1.2, or 2.4 mM) in the presence of SP600125 (10 μM), PD98059 (10 μM), and NAC (10 mM). **(E)** The transcriptional activity of p53 in dairy cows hepatocytes treated with different concentrations of NEFA (0, 0.3, 0.6, 1.2, or 2.4 mM) in the presence of SP600125 (10 μM), PD98059 (10 μM), and NAC (10 mM). **(F)** The NEFA-induced translocation of p53 from the cytosol to the nucleus in dairy cows hepatocytes incubated without or with NEFA (2.4 mM) in the presence of SP600125 (10 μM), PD98059 (10 μM), and NAC (10 mM). Hepatocytes were fixed, stained with a specific antibody against p53 (red fluorescence) and counterstained with Hoechst 33258 (blue fluorescence). All results were obtained from three independent experiments and are expressed as the mean ± SD. *p < 0.05 and **p < 0.01 vs. the control group.

P53 is a transcription factor that is sensitive to oxidative stress and that mediates apoptosis. Therefore, the transcriptional activity of p53 was determined via EMSA after hepatocytes were treated with NEFA in the absence or presence of the JNK inhibitor SP600125, the ERK inhibitor PD98059 or NAC. The protein and mRNA expression levels of p53 were determined via western blot and qRT-PCR. As shown in Figures 7A,B, NEFA significantly increased the protein expression levels of p53. Moreover, the JNK inhibitor SP600125 and NAC significantly inhibited the protein expression levels of p53, and the ERK inhibitor PD98059 enhanced the protein expression levels of p53 (Figures 7C,D). The results revealed that NEFA significantly promoted the transcriptional activity of p53 after NEFA treatment group (Figure 7E) but the JNK inhibitor SP600125 and NAC significantly decreased the NEFA-mediated stimulation of the transcriptional activity of p53, whereas the ERK inhibitor PD98059 further increased the transcriptional activity of p53. To further confirm that NEFA induces p53 translocation into the nucleus, immunofluorescence staining was performed. As shown in Figure 7F, NEFA markedly increased nuclear translocation of p53. Furthermore, the JNK inhibitor SP600125 and NAC significantly inhibited p53 nuclear translocation, and the ERK inhibitor PD98059 reduced p53 nuclear translocation. These results suggest that p53 is activated by NEFA via the ROS-ERK/JNK signaling pathway and participates in NEFA-induced dairy cow hepatocytes apoptosis.

### NEFA Inhibit the Transcriptional Activity, Translocation, and Expression of Nrf2 in Dairy Cow Hepatocytes

**FIGURE 8:**
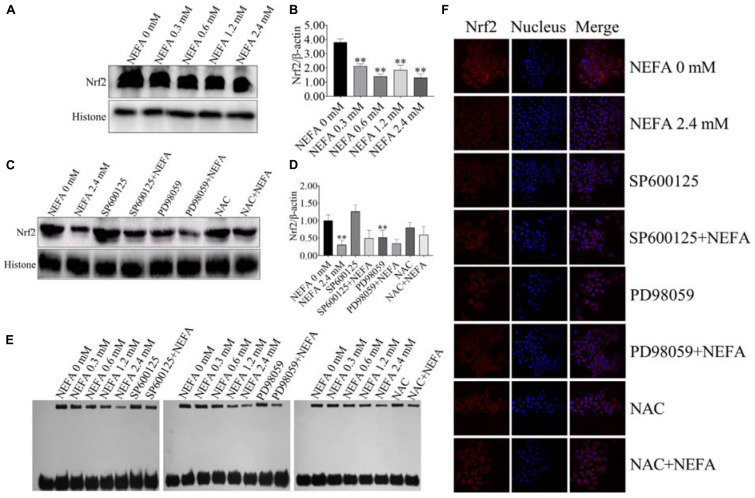
NEFA induce the inhibition of Nrf2 in dairy cow hepatocytes. **(A)** The protein expression levels and **(B)** quantification of Nrf2 in dairy cows heaptocytes incubated with different concentrations of NEFA (0, 0.3, 0.6, 1.2, or 2.4 mM). **(C)** The protein expression levels and **(D)** quantification of Nrf2 in dairy cows hepatocytes incubated with different concentrations of NEFA (0, 0.3, 0.6, 1.2, or 2.4 mM) in the presence of SP600125 (10 μM), PD98059 (10 μM), and NAC (10 mM). **(E)** The transcriptional activity of Nrf2 in dairy cows treated with different concentrations of NEFA (0, 0.3, 0.6, 1.2, or 2.4 mM) in the presence of SP600125 (10 μM), PD98059 (10 μM), and NAC (10 mM). The NEFA-induced translocation of Nrf2 from the cytosol to the nucleus in dairy cows hepatocytes incubated without or with NEFA (2.4 mM) in the absence or presence of SP600125 (10 μM), PD98059 (10 μM), and NAC (10 mM). Hepatocytes were fixed, stained with a specific antibody against Nrf2 (red fluorescence) and counterstained with Hoechst 33258 (blue fluorescence). All results were obtained from three independent experiments and are expressed as the mean ± SD. *p < 0.05 and **p < 0.01 vs. the control group.

To determine whether Nrf2 plays an important role in NEFA-induced apoptosis, hepatocytes were treated with various NEFA concentrations in the absence or presence of the two aforementioned inhibitors or NAC. NEFA decreased the protein expression levels of Nrf2 (Figures 8A,B). The JNK inhibitor SP600125 and NAC significantly increased the protein expression level of Nrf2, whereas the ERK inhibitor PD98059 further reduced the protein expression level of Nrf2 (Figures 8C,D). Then, the transcriptional activity of Nrf2 was determined via EMSA after treatment with NEFA in the absence or presence of the JNK inhibitor SP600125, the ERK inhibitor PD98059 or NAC. The results revealed that NEFA significantly inhibited the transcriptional activity of Nrf2 after NEFA treatment. As shown in Figure 8E, the JNK inhibitor SP600125 and NAC significantly increased the NEFA-inhibited transcriptional activity of Nrf2, whereas the ERK inhibitor PD98059 further inhibited the transcriptional activity of Nrf2. As shown in Figure 8F, Nrf2 translocation was determined via immunofluorescence. NEFA inhibited Nrf2 translocation from the cytosol into the nucleus. Furthermore, the JNK inhibitor SP600125 and NAC significantly increased Nrf2 translocation, whereas the ERK inhibitor PD98059 decreased the translocation of Nrf2 from the cytosol to the nuclear (Figure 8F). Taken together, these results demonstrate that NEFA inhibits the activation of Nrf2 via the ROS-ERK/JNK signal pathway and that Nrf2 participates in NEFA-induced dairy cow hepatocytes apoptosis.

## Discussion

Periparturient dairy cows are in a state of high metabolic status induced by pregnancy, parturition, the initiation of lactation and decreased dry matter intake. Ketosis is a common metabolic disease in dairy cows, and its pathologic basis is an NEB during the lactation period. Ketosis is characterized by hypoglycemia and high blood concentrations of NEFA and ketone bodies. Oxidative stress is involved in the development of certain metabolic diseases, such as human non-alcoholic fatty liver disease (NAFLD), diabetes and obesity.

ROS are the mediators of oxidative injury to cells. ROS serves both beneficial and deleterious roles in regulating intracellular signaling pathways (Suzuki et al., 1997). At low concentrations, ROS (such as H_2_O_2_ and O.^2–^) act as second messengers in signal transduction pathways. By contrast, excess ROS attack biomacromolecules, such as proteins, nucleic acid, and lipids, thereby inducing apoptosis (Marnett, 2000; Stadtman and Levine, 2000). NEFA can act as energy molecules that are oxidized in mitochondria and that produce ATP to provide energy, accompanied by the production of a large amount of ROS. In our study, dairy cow hepatocytes were treated with various NEFA concentrations. We found that NEFA treatment significantly increased the intracellular ROS and MDA contents and decreased the GSH content, resulting in a significant increase in the hepatocytes apoptosis rate. These *in vitro* data were consistent with *in vivo* results and confirmed our hypothesis that NEFA induced hepatocytes oxidative stress and apoptosis. NAC is an antioxidant that reduces the levels of ROS. Pretreatment of hepatocytes with NAC protected hepatocytes from NEFA-induced oxidative stress and apoptosis. Oxidative stress damages DNA, lipids and proteins in various cell types (Ravindran et al., 2011). Further studies have shown that apoptosis plays a role in NAFLD-mediated liver damage and the increased ALT/ASL ratio in AML 12 hepatocytes subjected to curcumin-induced apoptosis (Cacicedo et al., 2005; Zhou et al., 2009). Dairy cows with ketosis are in a state of NEB, resulting in a high metabolic state of NEFA in their hepatocytes. Larger amounts of ROS generated during the process of NEFA oxidation in mitochondria. ROS attack the hepatocytes cell membrane and biomacromolecules, subsequently causing oxidative injury, thereby aggravating ketosis in dairy cows.

Increased FFA metabolism leads to increased ROS production. ROS are essential for FFA-induced apoptosis (Sparagna et al., 2001). Our results demonstrated that the ROS levels and the apoptosis rate were increased in a NEFA dose-dependent manner in dairy cow hepatocytes. In the early stage of apoptosis, oxidative stress induces mitochondrial dysfunction, leading to cell death (Ravindran et al., 2011). NEFA, such as palmitic acid, are found to increase ROS production, inducing caspase- and mitochondrial dysfunction-mediated apoptosis (Cacicedo et al., 2005; Zhou et al., 2009). ROS causes apoptosis, organ damage and inflammation via the activation of several signaling pathways, including MAPK signaling pathways. The MAPK signaling pathways consist of three kinases, JNK, p38 MAPK, and ERK, which participate in cell proliferation, apoptosis and cell differentiation (Peverelli et al., 2010). The activation of MAPK signaling pathways increases caspase activity and induces mitochondrial dysfunction, which is involved in the progression of rat endplate chondrocyte apoptosis (Kong et al., 2013). A recent study revealed that p38 is involved in NEFA-induced dairy cow hepatocytes apoptosis (Song et al., 2014). However, whether JNK and ERK participate in NEFA-mediated hepatocytes apoptosis is unclear. In the present study, we found that phosphorylation levels of JNK were increased in a NEFA dose-dependent manner. Alternatively, the phosphorylation levels of ERK were decreased after NEFA treatment in dairy cow hepatocytes. These results suggested that JNK and ERK might be involved in NEFA-induced hepatocytes apoptosis. To further examine the role of JNK and ERK in NEFA-induced hepatocytes apoptosis, hepatocytes were pretreated with SP600125 and PD98059, respectively. SP600125 decreased NEFA-induced hepatocytes apoptosis, whereas PD98059 further increased dairy cow hepatocytes apoptosis. Taken together, our results suggest that JNK and ERK are involved in NEFA-induced dairy cow hepatocytes apoptosis. Furthermore, as a result of pretreatment with NAC, the phosphorylation levels of ERK were increased. However, JNK phosphorylation was decreased after the hepatocytes were pretreated with NAC. Together with our previous studies, these results demonstrated that NEFA-induced hepatocytes apoptosis is mediated by oxidative stress and the ROS-ERK/JNK signaling pathway.

Mitochondria play a crucial role in the transduction of apoptosis signaling via the intrinsic apoptosis pathway (Green and Reed, 1998). A loss of the MMP is an expression of mitochondrial damage, which leads to changes in mitochondrial permeability (Chen Q. et al., 2012). Additionally, the present study indicated that NEFA induced a loss of the MMP, ultimately leading to mitochondrial damage. Moreover, this effect was alleviated by SP600125 and NAC and exacerbated by PD98059. Pro-apoptotic signaling leads to the translocation of cyt *c* or AIF from the mitochondrial intermembrane space to the cytosol, which triggers the caspase-dependent or caspase-independent signaling pathway, respectively. Cytosolic cyt *c* binds to apaf-1, forming the apoptosome, which subsequently recruits caspase 9. Activated caspase 9 cleaves its downstream caspase 3 (Slee et al., 1999). AIF mediates nuclear chromatin condensation and DNA fragmentation via a caspase-independent pathway by translocating from the outer mitochondrial membrane to the cytosol (Daugas et al., 2000). Bcl-2 family proteins also play a key role in regulating the mitochondrial apoptotic pathway, primarily by modulating the MMP and the release of cyt *c* and other apoptotic factors. There are three groups of Bcl-2 family proteins: the prosurvival Bcl-2 proteins, such as Bcl-2, Bcl-xl, Bcl-w, Mcl-1, and A1, the executioners Bax and Bak and the BH3-only proteins, which inhibit cell death, directly participate in mitochondrial outer membrane (MOM) permeabilization and initiate apoptosis, respectively. However, in the present study, the protein expression of Bax, Bcl-2, AIF, and cyt *c* showed the expected changes after SP600125 (an inhibitor of JNK) and NAC (a ROS scavenger) treatment compared with NEFA alone. The protein expression of Bax, AIF and cyt *c* release decreased and the protein of Bcl-2 increased. These results were similar to the previous reports that Bax expression, cyt *c* release, and caspase-3 were induced by cisplatin followed by cell apoptosis which was prevented by MEK inhibitor (PD98059) (Kim et al., 2005). Our results also suggested that NEFA induced greater Bax, and AIF expression and cyt *c* release, reduced Bcl-2 expression, and the effects were prevented by the ERK inhibitor (PD98059). These results indicated that ERK reduction prevents cyt *c* release, AIF expression by inhibiting Bax expression, and thus acts upstream of the mitochondrial-dependent apoptotic pathway in NEFA-treatment hepatocytes. Activation of caspase 3 and PARP and protein expression of apaf-1 and ASK1 showed the expected changes after SP600125 (an inhibitor of JNK) and NAC (a ROS scavenger) treatment compared with NEFA alone. In the present study, the cleavage of caspase 3 and PARP and protein expressions of apaf-1 and ASK1 increased. These results were similar to previous reports that caspase-3 was induced by cisplatin followed by cell apoptosis which was prevented by MEK inhibitor (PD98059) (Kim et al., 2005). Our results also suggested that NEFA induced greater cleavage of caspase 3 and protein expressions of apaf-1 and ASK1 and the effects were prevented by the ERK inhibitor (PD98059). Combined with the above results, ERK reduction prevents cyt *c* release, the cleavage of caspase 3 and PARP and protein expressions of AIF, apaf-1, and ASK1 by inhibiting Bax expression and thus acts upstream of the mitochondrial-dependent apoptotic pathway in NEFA-treatment hepatocytes.

JNK and ERK are crucial signal transduction effectors that respond to a wide range of extracellular stresses, particularly oxidative stress. Activation of p38 MAPK affects the regulation of downstream transcription factors, such as p53 and Nrf2, to modulate the expression of downstream pro-apoptotic and anti-apoptotic genes (Song et al., 2014). However, few reports confirm whether JNK and ERK are involved in the regulation of p53 and Nrf2 in dairy cow hepatocytes. The redox-sensitive transcription factor p53 plays a critical role in FFA-induced apoptosis (Yuan et al., 2010). As a transcription factor, the tumor suppressor p53 transcriptionally activates the pro-apoptotic gene Bax and transcriptionally represses the anti-apoptotic gene Bcl-2, modulating the mitochondrial, or intrinsic, apoptosis pathway (Johnstone et al., 2002; Yee and Vousden, 2005). Also, p53 inhibits the transcriptional activation of Nrf2 target genes in many cell lines (Faraonio et al., 2006). Recent studies have suggested that the inhibition of Nrf2 is essential for the activation of the p53 apoptosis pathway (Iida et al., 2007; Chen W. et al., 2012). Moreover, the role of Nrf2 in apoptosis is mediated by its regulation of Bcl-2 family proteins (Chen Q. et al., 2012; Niture and Jaiswal, 2012). Therefore, we investigated the role of both p53 and Nrf2 in NEFA-treated dairy cow hepatocytes. Our results revealed that NEFA significantly increased the expression levels and transcriptional activity of p53 and significantly decreased the expression levels and transcriptional activity of Nrf2. These responses to NEFA in dairy cow hepatocytes were effectively reversed by pretreatment with an inhibitor (SP600125) and NAC. In contrast, PD98059 inhibited the transcriptional activity, translocation and expression levels of Nrf2 in the absence or presence of NEFA. These results are consistent with data from the literature indicating that the MAPKs JNK and ERK are the upstream kinases p53 and Nrf2 responsible for apoptosis in response to various stimuli and that p53 and Nrf2 are sensitive to oxidative stress (Piccirillo et al., 2009; Yuan et al., 2010; Runchel et al., 2011; Chen W. et al., 2012). These results indicate that ROS, JNK and ERK play crucial roles in the NEFA-induced alterations in the transcription factors p53 and Nrf2 in dairy cow hepatocytes. Together with our previous studies, these findings suggest that NEFA-induced dairy cow hepatocytes apoptotic damage is mediated by the ROS-p38-p53/Nrf2 pathway (Song et al., 2014). Overall, our results indicated that liver damage in cows with high concentrations of NEFA during the transition period is associated with the ROS-MAPK (p38, ERK, and JNK)-p53/Nrf2 apoptosis signaling pathway. Based on our findings, NAC and MAPK inhibitors (SP600125, PD98059, and SB203580) may protect dairy cows from liver damage. Therefore, we provide a theoretical basis for effectively preventing ketosis in dairy cows.

In summary, this study suggested that NEFA are capable of inducing oxidative stress-mediated damage, causing dairy cow hepatocytes apoptosis. The results of this study demonstrated that NEFA trigger hepatocytes apoptosis via the JNK/ERK activation-mediated mitochondria-dependent apoptosis pathway, including the transcriptional activation of p53, the transcriptional inhibition of Nrf2, a loss of the MMP, the cleavage of caspase 3 and PARP and the translocation of AIF and cyt *c* as crucial events involved in apoptosis. These findings indicate that the ROS/MAPK/mitochondrial apoptosis signaling pathway plays a crucial role in NEFA-induced hepatocytes damage.

## Conclusion

In conclusion, the high concentration of NEFA results in excessive ROS production, which activates the JNK pathway, inhibits the ERK pathway and leads to the transcriptional activation of p53 and the transcriptional inhibition of Nrf2. Excessive oxidative stress signals initiate hepatocytes apoptosis, ultimately causing liver damage.

## Data Availability Statement

All datasets generated for this study are included in the article/supplementary material.

## Ethics Statement

The dairy cows used in this study were approved for animal experiments and were conducted according to the Chinese law for the welfare of animals, and approved by the Anhui Agricultural University.

## Author Contributions

YL, HD, and GL designed the project. YL, HD, LL, YS, and SF performed the experiments and analyzed the results. XD, XW, XBL, ZW, XWL, JL, JW, and GL revised the manuscript. YL and HD drafted the manuscript. All authors revised the final version of the manuscript.

## Conflict of Interest

The authors declare that the research was conducted in the absence of any commercial or financial relationships that could be construed as a potential conflict of interest.
