# Corrigendum: Non-esterified Fatty Acid Induce Dairy Cow Hepatocytes Apoptosis via the Mitochondria-Mediated ROS-JNK/ERK Signaling Pathway

**DOI:** 10.3389/fcell.2020.00462

**Published:** 2020-06-19

**Authors:** Yu Li, Hongyan Ding, Leihong Liu, Yuxiang Song, Xiliang Du, Shibin Feng, Xichun Wang, Xiaobing Li, Zhe Wang, Xinwei Li, Jinchun Li, Jinjie Wu, Guowen Liu

**Affiliations:** ^1^College of Animal Science and Technology, Anhui Agricultural University, Hefei, China; ^2^Key Laboratory of Zoonosis, Ministry of Education, College of Veterinary Medicine, Jilin University, Changchun, China

**Keywords:** NEFA, apoptosis, mitochondria-mediated ROS-JNK/ERK signaling pathway, dairy cow, hepatocyte

In the original article, there was a mistake in Figures 7 and 8 as published. The word “Necueus” should be changed to “Nucleus.” We have also removed the red line in Figure 8.

**Figure 7:**
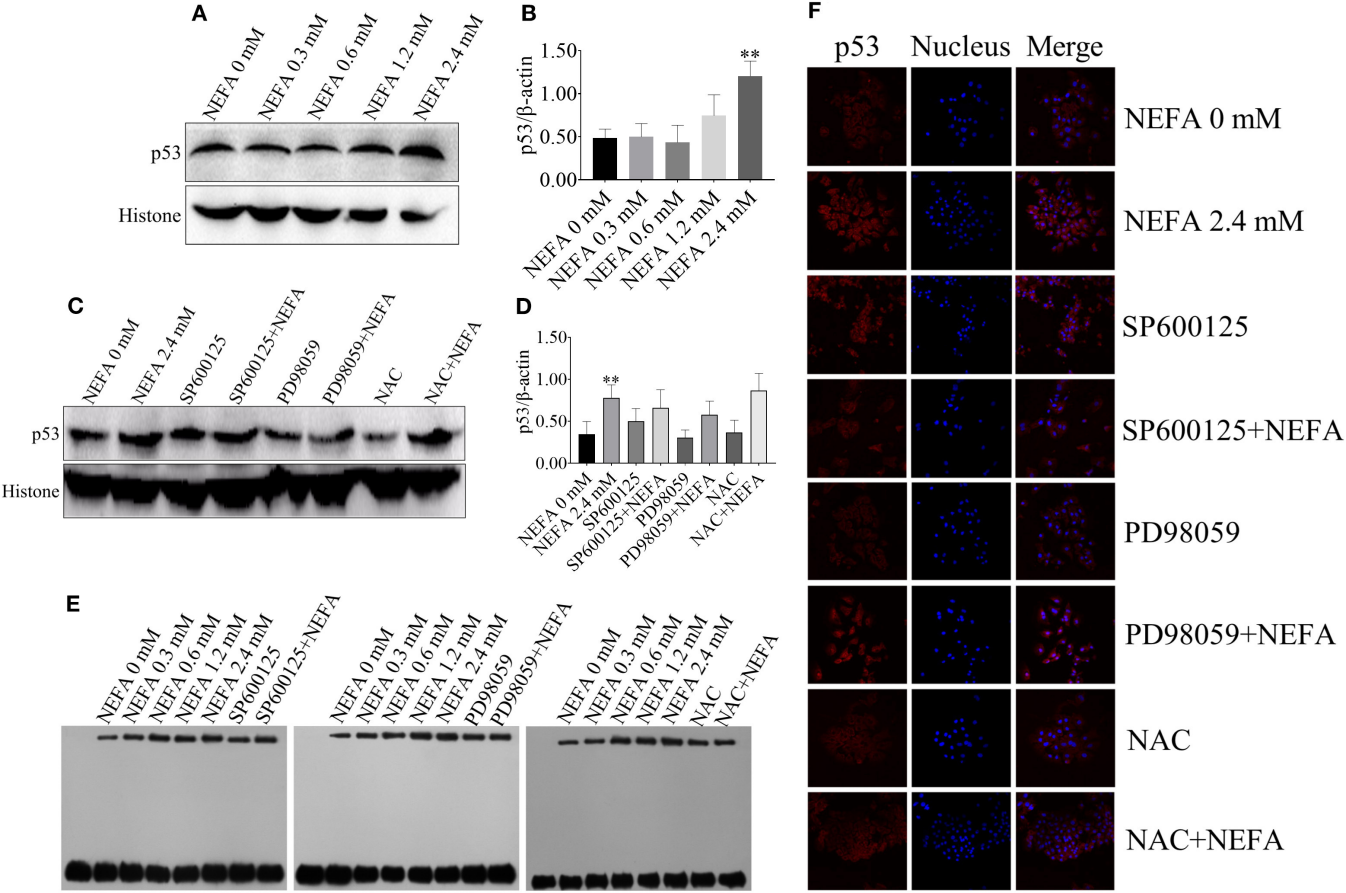
Effect of NEFA on the activation of p53. **(A)** The protein expression levels and **(B)** quantification of p53 in dairy cows heaptocytes incubated with different concentrations of NEFA (0, 0.3, 0.6, 1.2, or 2.4 mM). **(C)** The protein expression levels and **(D)** quantification of p53 in dairy cows heaptocytes incubated with different concentrations of NEFA (0, 0.3, 0.6, 1.2, or 2.4 mM) in the presence of SP600125 (10 μM), PD98059 (10 μM), and NAC (10 mM). **(E)** The transcriptional activity of p53 in dairy cows hepatocytes treated with different concentrations of NEFA (0, 0.3, 0.6, 1.2, or 2.4 mM) in the presence of SP600125 (10 μM), PD98059 (10 μM), and NAC (10 mM). **(F)** The NEFA-induced translocation of p53 from the cytosol to the nucleus in dairy cows hepatocytes incubated without or with NEFA (2.4 mM) in the presence of SP600125 (10 μM), PD98059 (10 μM), and NAC (10 mM). Hepatocytes were fixed, stained with a specific antibody against p53 (red fluorescence) and counterstained with Hoechst 33258 (blue fluorescence). All results were obtained from three independent experiments and are expressed as the mean ± SD. ^*^p < 0.05 and ^**^p < 0.01 vs. the control group.

**Figure 8:**
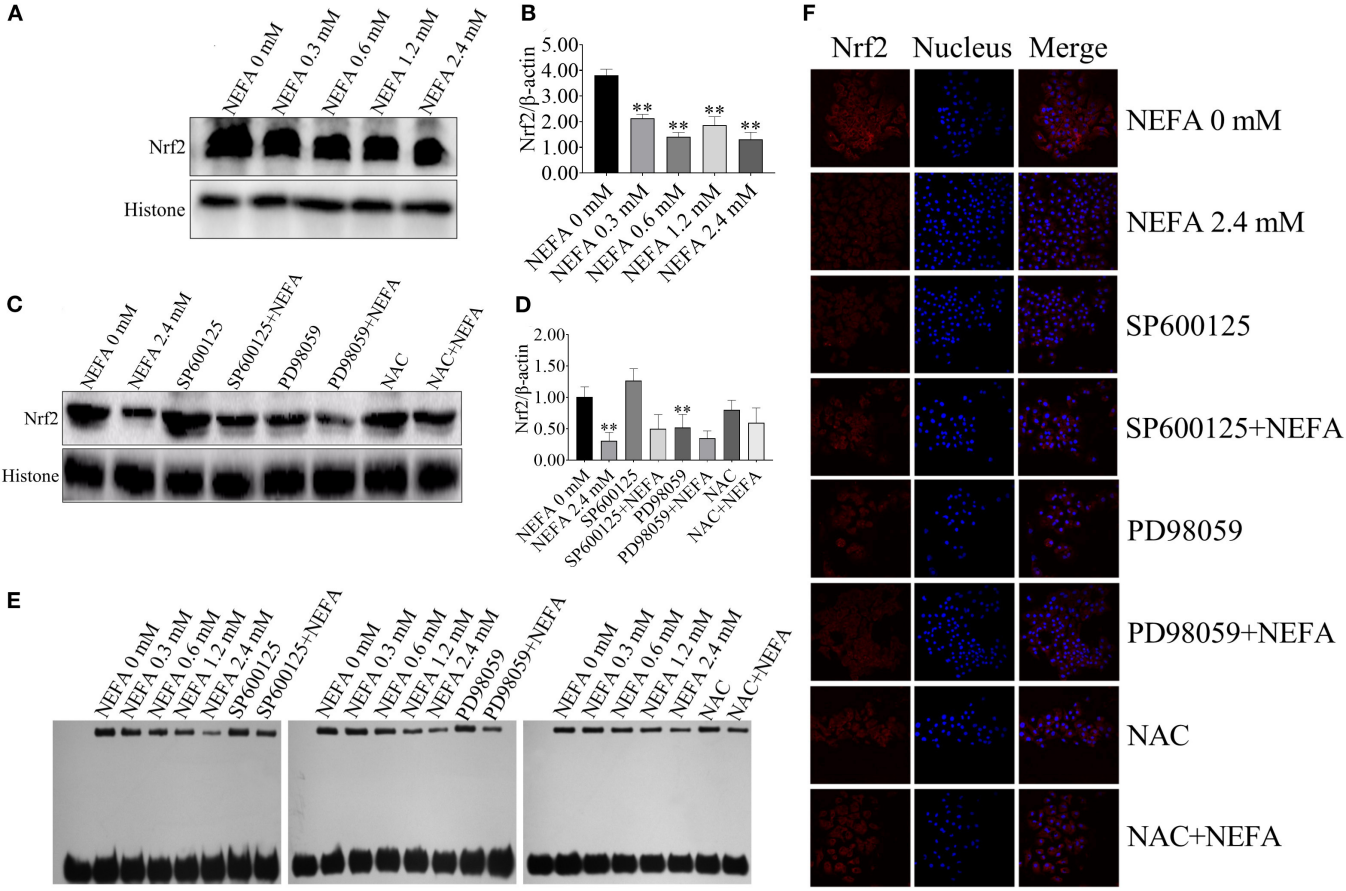
NEFA induce the inhibition of Nrf2 in dairy cow hepatocytes. **(A)** The protein expression levels and **(B)** quantification of Nrf2 in dairy cows heaptocytes incubated with different concentrations of NEFA (0, 0.3, 0.6, 1.2, or 2.4 mM). **(C)** The protein expression levels and **(D)** quantification of Nrf2 in dairy cows hepatocytes incubated with different concentrations of NEFA (0, 0.3, 0.6, 1.2, or 2.4 mM) in the presence of SP600125 (10 μM), PD98059 (10 μM), and NAC (10 mM). **(E)** The transcriptional activity of Nrf2 in dairy cows treated with different concentrations of NEFA (0, 0.3, 0.6, 1.2, or 2.4 mM) in the presence of SP600125 (10 μM), PD98059 (10 μM), and NAC (10 mM). **(F)** The NEFA-induced translocation of Nrf2 from the cytosol to the nucleus in dairy cows hepatocytes incubated without or with NEFA (2.4 mM) in the absence or presence of SP600125 (10 μM), PD98059 (10 μM), and NAC (10 mM). Hepatocytes were fixed, stained with a specific antibody against Nrf2 (red fluorescence) and counterstained with Hoechst 33258 (blue fluorescence). All results were obtained from three independent experiments and are expressed as the mean ± SD. *p < 0.05 and **p < 0.01 vs. the control group.

The authors apologize for this error and state that this does not change the scientific conclusions of the article in any way. The original article has been updated.

